# Eggshell calcification of the urinary bladder in chronic *Schistosoma haematobium* infection: A radiologic-pathologic case report

**DOI:** 10.1016/j.radcr.2026.06.021

**Published:** 2026-07-04

**Authors:** Juliana Andrade, Joana Sobreiro Silva

**Affiliations:** a Internal Medicine Department, Unidade Local de Saúde de Braga, Braga, Portugal; b Anatomic Pathology, Unidade Local de Saúde de Braga, Braga, Portugal

**Keywords:** *Schistosoma haematobium*, Urinary schistosomiasis, Eggshell bladder, Hematuria, Computed tomography, Case report

## Abstract

Chronic urinary schistosomiasis is an important cause of hematuria and genitourinary morbidity in individuals from endemic regions. In advanced disease, computed tomography (CT) may show circumferential calcification of the bladder and distal ureters, a classic pattern that can suggest the diagnosis even when laboratory tests are negative. A 38-year-old man from Mozambique, living in Portugal with human immunodeficiency virus and hepatitis B virus infections, presented with 2 months of painless gross hematuria. He denied dysuria, fever, flank pain, weight loss, prior urinary tract instrumentation, tuberculosis, or recent travel, and baseline blood tests and renal function were normal.

Urinalysis showed hematuria and leukocyturia, but multiple urine examinations for Schistosoma ova and a urine Schistosoma-specific polymerase chain reaction were negative. Noncontrast CT of the abdomen and pelvis was obtained and demonstrated diffuse circumferential calcification of the urinary bladder wall with distal ureteral involvement, producing a classic “eggshell” appearance without hydronephrosis, renal stones, or intraluminal mass. Cystoscopy showed yellowish plaques and nodular mucosal lesions, and targeted bladder biopsies revealed numerous calcified Schistosoma haematobium ova within the urothelium and submucosa with chronic inflammation and stromal calcification, confirming chronic inactive infection.

The patient received a single dose of praziquantel 40 mg/kg and 3 months later, hematuria had resolved and renal function was stable. This case highlights that in chronic urinary schistosomiasis, CT findings can be decisive when urine-based tests are negative.

## Introduction

Urinary schistosomiasis, most commonly caused by *Schistosoma haematobium*, remains a major cause of hematuria and chronic urogenital morbidity in endemic regions [1,2]. Chronic deposition of eggs in the bladder wall and distal ureters leads to granulomatous inflammation, fibrosis, and dystrophic calcification, which can be detected on imaging [[3], [4], [5]].

Radiologic findings in long-standing urinary schistosomiasis include bladder wall thickening, mucosal irregularity, distal ureteral involvement, and, in advanced disease, diffuse circumferential mural calcification producing the so-called “eggshell bladder” appearance [4,5]. In nonendemic settings, this pattern is particularly valuable because clinical suspicion may be low and urine microscopy or molecular testing may be negative in chronic disease [2,3,5].

This report describes a patient in whom urine microscopy and urine PCR were nondiagnostic, while CT provided the key diagnostic clue that led to cystoscopy and targeted biopsy. The main educational contribution of this case is therefore not the rarity of the imaging sign itself, but the demonstration that imaging may become decisive when routine laboratory evaluation fails to establish the diagnosis.

## Case presentation

A 38-year-old man originally from Mozambique and living in Portugal for several years, with known HIV and HBV infections under regular follow-up, presented with a 2-month history of painless gross hematuria. He denied dysuria, flank pain, fever, weight loss, recent travel to endemic areas, or prior urologic procedures, and there was no known history of urolithiasis or tuberculosis.

On physical examination, vital signs were stable and abdominal and genitourinary examinations were unremarkable. Baseline laboratory tests showed normal hemoglobin, leukocyte, and platelet counts, with preserved renal function. Urinalysis confirmed hematuria and leukocyturia without significant proteinuria.

Multiple urine specimens were examined for *Schistosoma* ova using standard microscopy, and all were negative. A *Schistosoma*-specific PCR assay on urine was also negative. Because hematuria persisted in an at-risk individual and the initial laboratory work-up was inconclusive, cross-sectional imaging was requested. Although ultrasonography is often used as an initial examination in endemic settings, CT was selected in this patient because it offers superior characterization of mural calcification and distal ureteral involvement, which were the main diagnostic considerations after nondiagnostic laboratory results [6].

Noncontrast CT of the abdomen and pelvis (Fig. 1) demonstrated striking circumferential calcification of the entire bladder wall, extending into both distal ureters, resulting in an “eggshell” pattern of mural calcification. Mild bladder wall thickening was present, but there was no intraluminal mass, hydronephrosis, or renal calculi. In the clinical and epidemiologic context, the radiologic appearance was considered highly suggestive of chronic urinary schistosomiasis despite the negative urine microscopy and PCR findings.

**Fig. 1:**
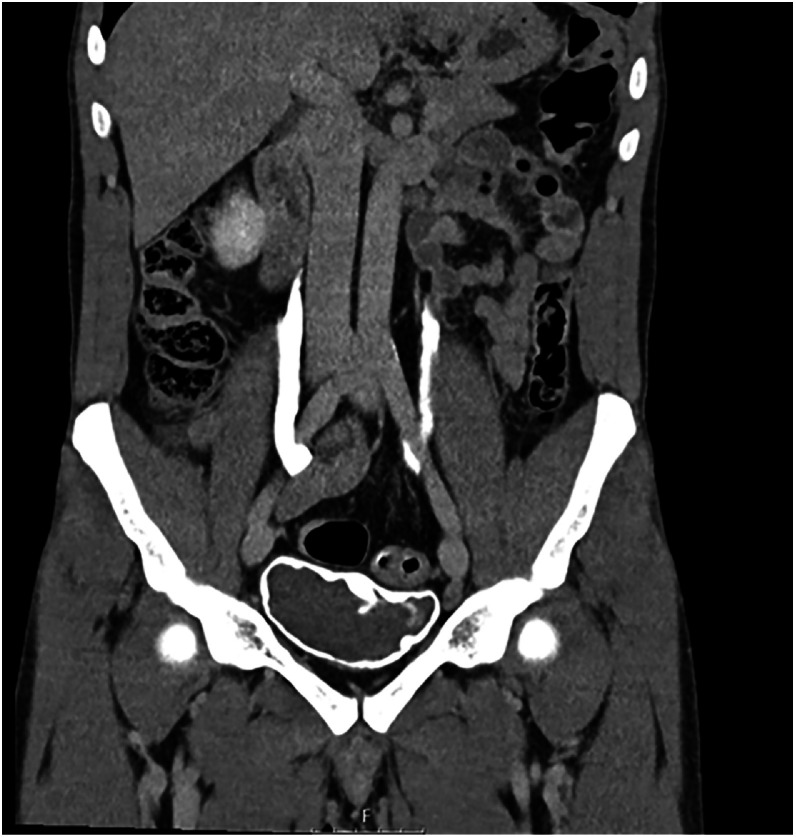
Noncontrast CT of the abdomen and pelvis, reviewed with multiplanar reformations; coronal soft-tissue reconstruction shown. The image demonstrates diffuse circumferential mural calcification of the urinary bladder, extending into both distal ureters, producing the characteristic “eggshell” appearance of chronic urinary schistosomiasis. Mild bladder wall thickening is present, with no intraluminal mass, hydronephrosis, or renal calculi.

Cystoscopy showed multiple irregular yellowish plaques and nodular lesions involving large areas of the bladder mucosa, with chronic inflammatory change and decreased bladder compliance. Targeted biopsies of these areas were obtained.

Histopathologic examination revealed numerous calcified *S haematobium* ova embedded in the urothelium and submucosa, surrounded by chronic inflammatory infiltrates and extensive stromal calcification. No dysplasia or malignancy was identified (Fig. 2). These findings confirmed chronic urinary schistosomiasis with calcified tissue eggs, indicating longstanding inactive disease rather than active transformation of eggs into other parasitic stages.

**Fig. 2:**
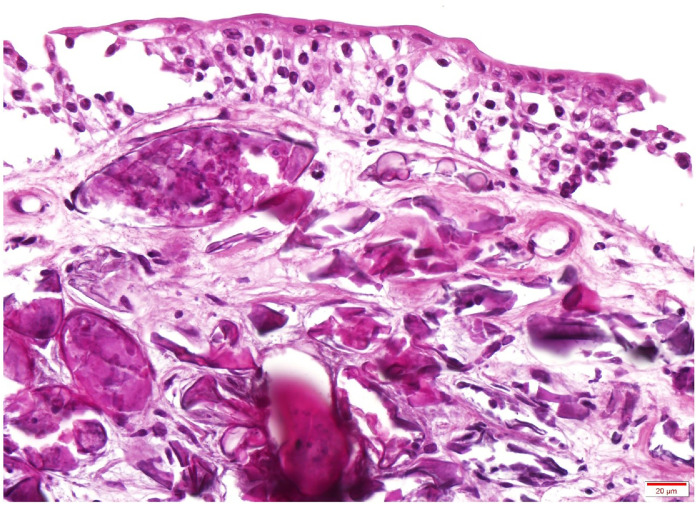
Histopathologic section of the bladder wall showing numerous calcified *Schistosoma haematobium* ova within the urothelium and submucosa, with chronic inflammatory infiltrate and stromal calcification on hematoxylin and eosin staining; scale bar = 20 µm.

The patient was treated with praziquantel 40 mg/kg as a single dose and counseled regarding possible long-term complications, including obstruction and bladder malignancy risk. At 3-month follow-up, hematuria had completely resolved, renal function remained normal, and no new urinary symptoms were reported. No follow-up imaging had yet been performed at the time of writing, but clinical and urologic follow-up was planned. The key clinical events from symptom onset to follow-up are summarized in Table 1.

**Table 1:** Clinical timeline from symptom onset to three-month follow-up.

Main events	Clinical event
Two months before evaluation	Onset of painless gross hematuria
Initial evaluation	Stable examination; normal blood counts; preserved renal function; urinalysis with hematuria and leukocyturia
Diagnostic laboratory testing	Multiple urine microscopy samples negative for *Schistosoma* ova; urine PCR negative
Imaging	Noncontrast CT showed circumferential bladder wall and distal ureteral calcification with classic “eggshell” appearance
Endoscopic assessment	Cystoscopy demonstrated yellowish plaques and nodular mucosal lesions
Histopathology	Calcified *S. haematobium* ova in the urothelium and submucosa with chronic inflammation and stromal calcification
Treatment	Praziquantel 40 mg/kg single dose
Follow-up	Complete resolution of hematuria at 3 months; renal function remained normal

## Discussion

The principal educational message of this case is that the diagnosis became possible only after imaging disclosed a characteristic pattern that was not captured by urine-based tests. Although “eggshell” bladder calcification is a classic imaging manifestation of chronic urinary schistosomiasis, its value in nonendemic practice lies in triggering the correct diagnostic pathway when microbiologic and molecular studies are negative. In this patient, CT redirected the work-up toward cystoscopy and biopsy, which ultimately established the diagnosis.

The case shows the practical diagnostic importance of a classic sign in a clinically challenging context, namely a patient with hematuria, epidemiologic risk, and negative urine microscopy and PCR. That sequence is educational for radiologists and clinicians practicing in nonendemic settings, where schistosomiasis may not be considered early in the differential diagnosis.

The differential diagnosis of bladder wall calcification includes genitourinary tuberculosis, encrusted cystitis, amyloidosis, prior intravesical chemotherapy or radiotherapy, and metastatic calcification related to chronic renal failure [7]. In the present case, several features supported schistosomiasis over these alternatives: the calcification was diffuse, symmetric, and circumferential; both distal ureters were involved; the patient originated from an endemic country; there was no history of prior intravesical therapy or pelvic radiotherapy; renal function was preserved; and there were no clinical data suggesting chronic renal failure or active tuberculosis.

Genitourinary tuberculosis more often produces irregular bladder wall thickening, fibrosis, ureteral strictures, contracted bladder, and upper tract abnormalities rather than a smooth circumferential “eggshell” calcified pattern. Encrusted cystitis is usually associated with severe irritative urinary symptoms, alkaline urine, and superficial calcified plaques related to urea-splitting organisms, which was less compatible with this presentation of painless gross hematuria and diffuse mural calcification [7]. Thus, the combination of imaging morphology and epidemiologic context substantially narrowed the differential diagnosis even before tissue confirmation.

Although ultrasonography is widely used as an initial imaging method in endemic settings and can identify bladder wall thickening, polypoid lesions, and distal ureteral abnormalities, CT was considered more informative here because the key unresolved question after negative urine tests was whether there was mural calcification or another structural explanation for hematuria. CT therefore had the highest immediate diagnostic yield in this particular clinical scenario.

Previous authors have even questioned whether HIV infection might accelerate the long-term complications of chronic schistosomiasis, but this issue remains to be elucidated [8]. Accordingly, in our patient, HIV co-infection was considered an important comorbidity and a source of diagnostic complexity, yet the decisive elements for establishing the final diagnosis were the characteristic CT findings and histopathologic confirmation rather than any HIV-related modification of the imaging pattern.

Calcified schistosomal eggs embedded in the bladder wall represent chronic tissue deposition and do not transform into miracidia or cercariae; accordingly, the mural calcification seen on CT should be understood as evidence of long-standing inactive tissue disease rather than a radiologic marker of parasitic reactivation. This distinction is relevant when interpreting apparently discordant results such as extensive calcification with negative urine microscopy and PCR.

Praziquantel remains the treatment of choice because it reduces adult worm burden and prevents further egg deposition. However, established fibrosis and calcification are often irreversible, so patients may remain at risk for later complications such as ureteral obstruction and bladder malignancy and should undergo continued urologic follow-up. In this case, short-term clinical follow-up showed complete resolution of hematuria, but longer surveillance remained advisable.

## Conclusion

Diffuse circumferential calcification of the urinary bladder and distal ureters on CT is a classic manifestation of chronic *Schistosoma haematobium* infection. In patients from endemic regions presenting with hematuria, this imaging pattern should strongly raise suspicion for urinary schistosomiasis even when urine microscopy and PCR are negative. This case demonstrates that, in chronic disease, imaging may provide the decisive clue that leads to cystoscopy, histopathologic confirmation, appropriate treatment, and long-term surveillance.

## Authors' contributions

Juliana Andrade wrote the main manuscript text; Both authors collected and prepared the images; all authors reviewed the manuscript.

## Patient consent

Written informed consent for publication of this case report and associated images was obtained from the patient.
